# A Plea for General Education of Subjects of Hygiene

**Published:** 1881-11

**Authors:** 


### A PLEA FOR GENERAL EDUCATION ON SUBJECTS OF HYGIENE.

From an article on "Physical Culture and Mental Attainments," published in the *Sanitary News*, by Dr. J. G. Reed, we take the following:—

"Know thyself," is a law of holy writ as imperative as any mandate coming from that inspired source. The relative value of different kinds of knowledge is seen in instances in which adornment takes the place of usefulness, and modern notions of elegance supplant the convictions of duty and labor. A young lady of eighteen is master of several languages, can play all the instruments, from the piano to the harp, sing an opera with thrilling effect, rehearse the romances of the day, and faint in an astonishing manner, yet, when overtaken by sickness, how ridiculously ignorant she appears when she places her hand below her diaphragm to indicate the location of her lungs!

The remedy for our existing evils is education, and education of a kind which will put the people in possession of a knowledge of the laws of life and of health, and that will enable the young lady of the world to know the difference between pneumonia and the stomach ache.

